# Equipment Became Better in Backcountry Skiing—Did Severity of Injuries Decrease? An Analysis from the Swiss Alps

**DOI:** 10.3390/ijerph17030901

**Published:** 2020-02-01

**Authors:** Benedikt Gasser

**Affiliations:** Swiss Registries and Data Linkage - Swiss RDL, University of Bern, 3012 Berne, Switzerland; benedikt.gasser@yahoo.com; Tel.: +41-788-170-711

**Keywords:** Switzerland, NACA Score, severity of injury, backcountry skiing

## Abstract

*Background:* Large technical developments in avalanche transceivers as well as in ski–shoe-binding units should make backcountry skiing a safer sport and as a consequence, yield to a decrease in the number and severity of mountain emergency events. *Methods:* From 2009–2018, a total of 3044 mountain emergencies (953 females and 2091 males) were identified from the SAC (Swiss Alpine Club) central registry while backcountry skiing. These were classified descriptively by cause, whereby the severity of the mountain emergency was quantified with a NACA-Score (National Advisory Committee for Aeronautics Score). *Results:* A total of 1357 falls (44.6%), 558 emergencies caused by avalanches (18.3%), 408 cases of blocking (13.4%), 214 cases of illnesses (7.0%), 202 cases of losing way (6.6%), 138 cases of a crevasse accident (4.5%), and material failure in 30 cases (1%) were registered. For the remaining 137 cases (4.5%), no classification or rare forms were detected. No substantial sex differences were found in severity of injury, however looking at the two endpoints of the observed time frame, a significant increase in NACA-Score from 2009 to 2018 (2.1 ± 1.8 up to 2.6 ± 2.1, *p* < 0.01) was detected. *Conclusions:* The increase in the severity of mountain emergencies while backcountry skiing in the last decade might be due to the fact that too many inexperienced absolve backcountry tours. The tendency might be promoted by the improved material in the way that it seems easier to absolve a tour while underestimating potential hazards.

## 1. Introduction

Backcountry skiing has become increasingly popular and it is estimated that there are about 200,000–300,000 persons per year that are active in Switzerland [1,2,3,4]. In the Swiss Alps, with its current climatic conditions, it is possible to make backcountry tours in the period from November to April.

Backcountry skiing represents a combination of an endurance element and, in particular, for descent, a motoric coordinative element with eccentric muscle activity [5,6,7,8,9,10,11,12,13]. In addition to the physical challenges of this sport, the psychological dimension of backcountry skiing has to be mentioned, which includes the important ability to adequately assess the technical situation with the objective (avalanche situation, rockfall, weather change, etc.) [14] and subjective dangers (group dynamics, overestimation of physical possibilities, etc.) [15,16,17]. Aside from the positive aspects, risk of injury needs to be mentioned. Often, avalanche accidents are the main focus of attention in the discussion of risks and dangers [3,18]. This is not wrong, as deathly accidents often belong to this class [3,18]. However, other reasons such as falls or getting lost also have high relevance for backcountry skiing [3]. In addition, the technical development of a ski–shoe binding unit and technical gadgets in the last few years have taken place [16,19]. For example, in the 1990s, avalanche transceivers were difficult to use and a quick find was only possible for very experienced users [16]. However, this has changed significantly, and avalanche transceivers are nowadays easy to use by beginners with little practice, so consequently, deathly emergencies should be less frequent [16]. Focusing on the people that are active backcountry skiers, it is necessary to mention that due to this technical progress, it is possible that broader parts of society can potentially be reached [4]. This opens up the sport, particularly for the elderly, who are at higher risks for emergencies such as falls due to loss of postural stability or higher prevalence rates of cardiovascular affections such as sudden cardiac death [4,17,18,19,20,21,22,23,24]. In addition, hints exist that young men, in particular, take higher risks, whereas women seem to be generally more careful [4,25,26].

From the above-mentioned, it can be suggested that avalanche accidents should be less frequent and less severe due to technical developments alone. The same applies to other causes of accidents on backcountry tours such as falls or getting lost in foggy conditions, which should also be less common due to technical developments such as GPS (Global Positioning System), making orientation easier. Furthermore, the extreme development of the ski–shoe-binding unit for backcountry skis should also result in lower injury rates [3,27,28]. Nowadays, modern plate bindings of backcountry skis have a safety mechanism that has been present in alpine skiing for years [3,27,28]. The modern pin bindings of backcountry skis with inserts in the front of the shoe also have a security mechanism [3,27,28]. Despite these positive developments, literature regarding injury patterns and severity in backcountry skiing injuries is scarce for Switzerland. 

This leads to the aim of the present study to analyze the pattern of emergencies of backcountry skiing during the last ten years in the Swiss Alps. As hypotheses with potential falsification, it is postulated that (i) the number of mountain emergencies has not changed over the past decade, (ii) that the severity of events has not changed in the last ten years; and (iii) no link exists between age and the severity of mountain emergencies [29]. 

## 2. Material & Methods

### 2.1. Analyzed Population

For the analysis, all mountain emergency cases of the SAC central register for the period 2009 to 2018 while backcountry skiing were analyzed. The central register contains data from the Swiss Air Rescue Service (REGA), Air Glaciers Lauterbrunnen, Air Glaciers Sanenland, Register SAC, KWRO (Kantonale Walliser Rettungsorganisation), Snow and Avalanche Research Institute Davos, and the cantonal police registers. During this period, 3044 (953 female and 2091 male) people were rescued or salvaged by the mountain rescue service in the Swiss Alps while backcountry skiing. The term “mountain emergency” covers all events where mountaineers claim the help of mountain rescue services, or are affected by subjective and objective mountain hazards [3]. This also applies to illnesses and evacuations of uninjured mountaineers. Each mountain emergency included emergency number used, date, rescue organization, event, place, canton, activity, NACA-Score (National Advisory Committee for Aeronautics Score), nationality, birth date, sex, place of residence, coordinates, and a short report (Table 1) [30].

### 2.2. Data Preparation

In a first step, a classification was made into different categories of causes of mountain emergency cases (falls, blocking, losing way, avalanches, illnesses, other). This classification was originally developed by SAC to enable comparisons of all disciplines of mountaineering such as hiking, backcountry skiing, climbing, or classic mountaineering. The classification scheme was unique, meaning that no multiple classification was allowed. This was followed by a detailed data analysis for the missing entries. As failures of less than 5% hardly affected the validity of statements (for example, there was less than 5% of missing values for age) neglecting cases was allowed. For some analyses, a substitution method using a simple procedure (mean value imputation) was applied for the further statistical analyses [31,32]. 

### 2.3. Statistical Analyses

Descriptive statistics were calculated per calendar year for age and NACA Scores. Sex differences were analyzed for sub-classes and the whole sample for average age and average NACA Score with two-tailed heteroscedastic t-tests, whereby significance was attributed on an alpha = 0.05 level. To analyze changes over the ten-year observation period, linear regressions with calculation of the degree of determination (R^2^) were calculated. For the detected increase of NACA over the observational period for the whole sample, an unpaired two-tailed heteroscedastic t-test was conducted and calculation of effect sizes for all events and subgroups for the period starting 2009–2018 [33]. In addition, to analyze the relationship of age and NACA, linear regressions were separated for the subgroups by calculating the degree of determination (R^2^) of the form NACA_i_ = α × age_i_ + ε_i_ calculated. Furthermore, logistic regressions with the binary outcome NACA Score = 0 or NACA Score ≥ 1 were additionally calculated as NACA_i_ = α × age_i_ + ε_i_, allowing the odds-ratios to be calculated [33]. In order to calculate the development of risk aversion over time, the quantification method proposed by Arrow and Pratt was calculated [34]. Calculations were made with Microsoft Excel (Microsoft Inc., Redmond, WA, USA) and GraphPad Prism (GraphPad Software, Inc., La Jolla, CA, USA).

## 3. Results

**Figure 1:**
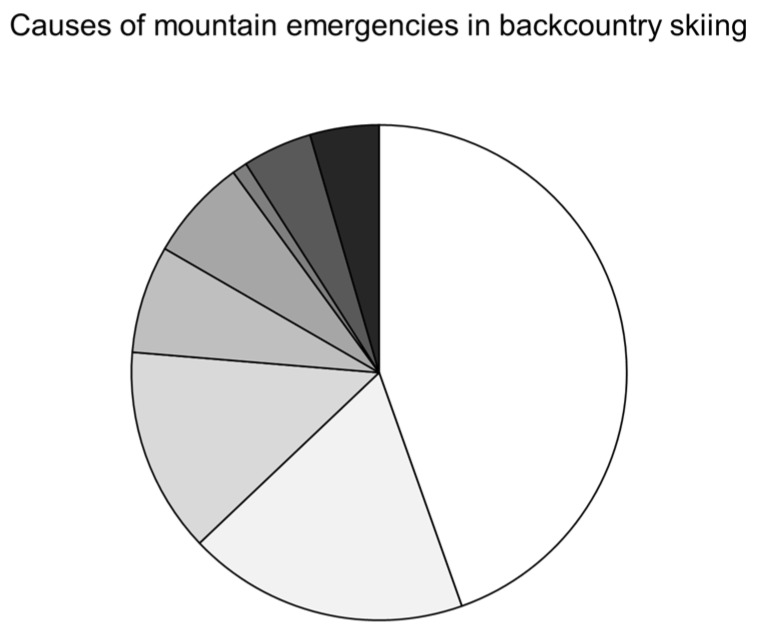
Causes of mountain emergencies in backcountry skiing (absolute and relative frequencies for the total sample and separated for female and male).

Figure 1 descriptively shows the different causes of mountain emergencies while backcountry skiing: 1357 falls (44.6%), followed by 558 cases of avalanches (18.3%), 408 cases (13.4%) of blocking, 214 cases (7.0%) of diseases, 202 cases (6.6%) of getting lost, 138 cases (4.5%) of crevasse accidents, and material failure in 30 cases (1%). For the remaining 137 cases (4.5%), there was no classification or very rare forms (sometimes one to two per calendar year) such as lightning/electric shock; hanging (rope); and crushing/pinching. Table 2 shows absolute frequencies of the different causes leading to mountain emergencies in backcountry skiing within the time period of 2009–2018 and includes diseases, falls, blocking, avalanches, getting lost, and all events across the different age groups. Concerning sex differences in total, around one third (n = 956) of mountain emergency cases were female and 68.6% were males (n = 2091). Two-tailed heteroscedastic t-tests concerning differences in age and NACA-Scores for sexes were conducted. However, there were no sex specific differences regarding the NACA-Score between females (2.3 ± 1.5) and males (2.3 ± 2.1). Mean age was significantly lower (*p* = 0.0403) within females (49.8 ± 12.3 years) than males (51.1 ± 12.3 years).

Concerning hypothesis (i), where the number of mountain emergencies has not changed over the past decade, Table 2 can be consulted, which shows events per subcategory. Taking all cases, an increase from 258 to 369 can be detected, however, the trend was not significant (*p* = 0.014). Furthermore, the estimated average annual increase was [1 − (369/258)^0.1^] × 100 = 3.5% per year, which was almost the same as the increase in the members in the Swiss Alpine Club during the respective time (around 4%) [35].

**Figure 2:**
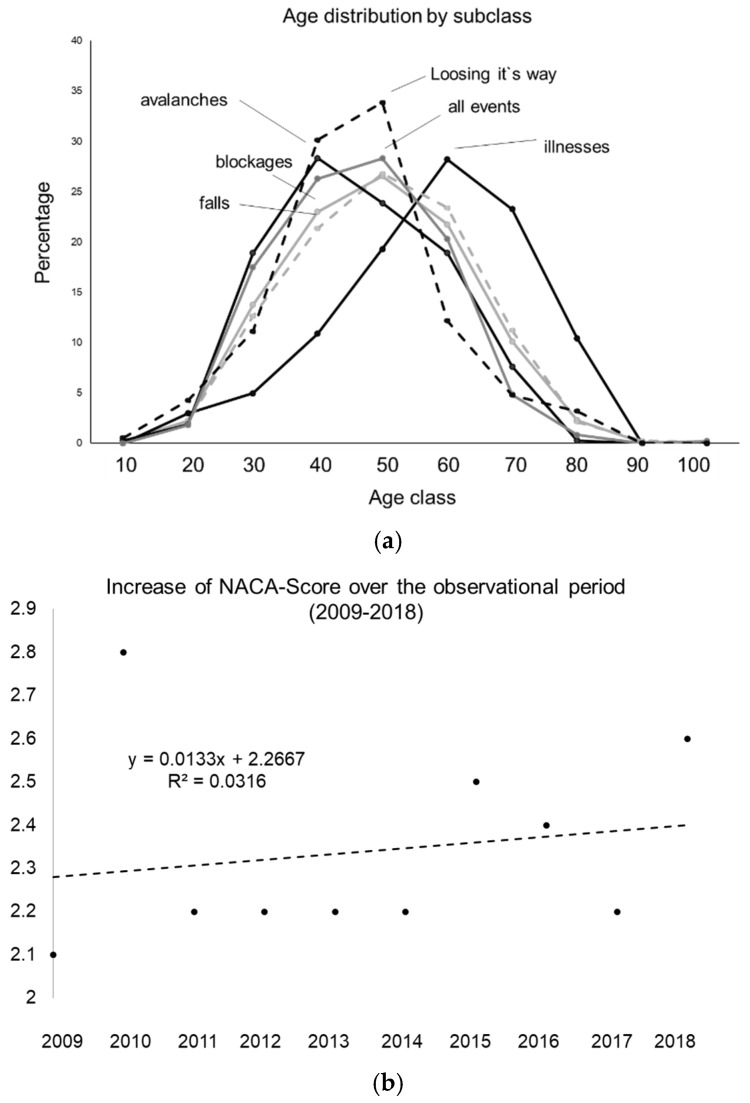
(**a**) A percentage age distribution of the shares in the respective classes. (**b**) The development of the NACA-Score over the observational period.

Concerning questioning hypothesis (ii), where the severity of events has not changed in the last ten years, linear regressions over time were calculated for the NACA-Scores (Figure 2b; Table 3). The regressions indicate an increase in the severity of mountain emergencies over the 10-year period over all events. However, only a small coefficient of determination of 0.0316 was identified. Average NACA Score increased highly significantly from 2.1 ± 1.8 in 2009 to 2.6 ± 2.1 in 2018 (*p* = 0.002), however, the calculated effect size over all events was 0.263, which was relatively small. Thereby, the big variance when looking at events per year has to be kept in mind. As a consequence, linear regressions were calculated over time for all events with NACA minus SD as the dependent variable yielding to y = 0.0248x + 0.2933 (R^2^ = 0.1692) and average NACA plus SD yielding to y = 0.0018x + 4.24 (R^2^ = 0.00002), with the addition of a low degree of coefficient of determination. As an indicator of a potential decrease, the respective increase in the severity risk aversion was calculated. The development of risk aversion was therefore tried for quantification with the concept of Arrow and Pratt. An estimation of average NACA on time (Table 3) was calculated, yielding an estimated equation: f(t) = 2.25 × t × 0.0229 (whereby t is the observational period from 2009–2018). This allows us to calculate the absolute risk aversion (AAR): − f′(t)/f″(t) for t_0_ = 2.1 and t_10_ = 2.6, yielding 0.465 and 0.376, respectively. As negative values imply risk pleasure, and positive values risk aversion in the original version, it is easily detectable that risk aversion decreases over time with an average rate by year, calculated as: 100 × {1 − [AAR (t_0_)/AAR (t_10_)]} = 2.16% per year, which would correlate with the premise of an increase in the severity of events. 

Concerning hypothesis (iii), that there is no link between age and severity of mountain emergencies, it is worth mentioning that linear regressions were calculated between age and NACA over the whole period in the form NACA_i_ = α × age_i_ + ε_i_ and logistic regressions with the binary outcome NACA Score = 0 or NACA Score ≥ 1. However, regression weights were not significant except for illnesses with α = −0.0001 for linear (*p* < 0.001), however, logistic (*p* = 0.7607) was not significant. For blocking, the regression weights were also very small (α < 0.0001 (*p* = 0.063) for the linear as well as for the logistic (*p* = 0.011). Furthermore, Figure 2 shows the age distribution of the shares in the respective classes. There seems to be a right shift in the illnesses (black) and a left shift in the avalanche congruent pattern with the total of all mountain accidents when backcountry skiing (grey).

## 4. Discussion

The aim of this study was to analyze the different causes of mountain emergencies in backcountry skiing. Concerning the premise that the number of mountain emergencies has not changed over the past decade, it is worth mentioning that on average, 309 ± 37 injured persons were reported per year, somewhat higher than the annually detected 268 persons in Austria [36]. Furthermore, the pattern of injury seems similar. In Austria, the biggest subclass of non-fatal injuries was also falls, however, with a slightly higher share of around 61% in Austria compared to the detected 44.6 in Switzerland [36]. Small injuries treated by self-confinement are not captured in the registry, counting for another part of the gap. However, cross-comparison with alpine skiing remains vague given the mentioned limitations [4,37]. To sum up, the cases (Table 1) increased by just under 4% per year, which was almost the same as the increase in SAC members during the respective time, which may be used as a proxy of activity, and therefore indicates that no real increase exists in the last decade [22,35,37]. Furthermore, the disposal of hypothesis (i) is especially challenged by the subclass of avalanches and getting lost. This might be due to different reasons. Preventive measures have had a high priority, especially for avalanches [37,38]. Current avalanche and snow conditions are nowadays easily available at a high level via the Internet (www.slf.ch) [38]. Furthermore, environmental factors such as climatic warming could also play a role, which, on average has led to less snowy winters with correspondingly fewer dangerous days [39]. Second, improved technology with better options for tour planning, and especially modern GPS, need to be mentioned as potentially having a preventive effect. Hypothesis (ii), according to which the severity of the events has not changed in the last ten years, seems vague when looking at the coefficient of determination, indicating a small variance explanation concerning an increase in the severity of events (Table 3). Furthermore, the validity of the results was challenged by the used NACA Score. Extensive analysis has shown that quantification of the severity of an injury can be difficult and large differences between individuals exist, which reduces the validity of results as a consequence [30]. Nevertheless, for the three subsamples of blocking, falls, and getting lost, the coefficient of determination was from 0.2 up to 0.53, indicating some explanation power indicating that over the observation period, the severity of mountain emergencies increased for these subclasses. This is astonishing, as the equipment has improved significantly in the last ten years with the potential to reduce the severity of emergency events. To sum up, the relatively general assumption that increasingly less experienced skiers may also be due to the good material to absolve backcountry tours may be dared. These might more often have an insufficiently trained safety sensorium with too offensive tour strategies predisposing the risk of severe injuries, which would be in line with the calculated decrease in risk aversion over the observational period [16,34].

Hypothesis (iii), that age has no explanatory power, seems partially disposable in line with the findings from others [18,19,20,21,22,23,24]. Aside from illnesses (mostly cardiovascular from in depth analysis of emergency case descriptions), falls were also somewhat more likely in the elderly, in line with the large problem of falls in the geriatric setting (Figure 2) [18,19,20,21,22,23,24]. As a counter-pool to diseases, the subclass of avalanches can be mentioned, which are more common in younger backcountry skiers (Figure 2a). Summing up in terms of age: the fact that avalanches are more common in younger people and the illnesses of older backcountry skiers suggests that there is a tendency for lower risk aversion in younger backcountry skiers, which in line with the findings from others [25,26,34].

## 5. Conclusions

No clear hints for a decrease in the number and severity of mountain emergencies while backcountry skiing can be detected. This is likely to suggest that in the last decade, too many unexperienced persons have absolved backcountry tours, which would be in line with the suggested decrease of risk aversion over the period. This tendency might be promoted by the improved material in the way that it seems easier to absolve a tour while underestimating the real difficulty. Careful tour planning with a serious evaluation of risks using a security margin is therefore recommended.

## Figures and Tables

**Table 1:** NACA-Score (National Advisory Committee for Aeronautics Score) [30].

Score	Description
NACA 0	No injury or disease. For example wrong alarm call
NACA I	Minor disturbance. No medical intervention is required. For example, slight abrasion.
NACA II	Slight to moderate disturbance. Outpatient medical investigation, but usually no emergency medical measures are necessary. For example, fracture of a finger bone, moderate cuts, moderate dehydration.
NACA III	Moderate to severe but not life-threatening disorder. Stationary treatment required, often emergency medical measures on the site. For example, femur fracture, milder stroke, smoke inhalation.
NACA IV	Serious incident where rapid development into a life-threatening condition cannot be excluded. In the majority of cases, emergency medical care is required. For example, vertebral injury with neurological deficit, severe asthma attack; drug poisoning.
NACA V	Acute danger. For example, third grade skull or brain trauma, severe heart attack.
NACA VI	Respiratory and or cardiac arrest.
NACA VII	Death

**Table 2:** Events per subcategory and calendar year over the 2009–2018 period.

	All Events	Blocking	Losing Way	Illnesses	Falls	Avalanches
2009	258	30	33	15	110	46
2010	330	40	25	18	119	93
2011	281	35	26	21	110	55
2012	260	42	20	12	120	32
2013	349	54	22	26	140	78
2014	314	46	16	25	154	107
2015	320	31	25	23	138	67
2016	324	40	16	33	145	56
2017	281	27	4	19	150	36
2018	369	60	15	22	167	57
Average per year	309 ± 37	41 ± 11	20 ± 8	21 ± 6	135 ± 20	63 ± 24
Coefficient of determination	0.2486	0.0798	0.6494	0.2498	0.825	0.0151
B-estimator	6.15	0.0798	−2.121	0.981	5.897	−0.9758

**Table 3:** Average NACA Scores over the whole period 2009–2018.

	All Events	Blocking	Falls	Loosing Way	Illnesses	Avalanches
	Mean ± SD	Mean ± SD	Mean ± SD	Mean ± SD	Mean ± SD	Mean ± SD
2009	2.1 ± 1.8	0.1 ± 0.4	2.7 ± 1	0.1 ± 0.4	3.1 ± 1.5	2.6 ± 2.8
2010	2.8 ± 2.1	0.2 ± 0.5	2.6 ± 0.9	0.04 ± 0.2	4.2 ± 2.0	3.2 ± 2.8
2011	2.2 ± 2.1	0.1 ± 0.4	2.8 ± 1	0.7 ± 2	3.7 ± 2	3 ± 3
2012	2.2 ± 1.9	0.1 ± 2.1	2.6 ± 1.5	0.1 ± 0.2	3.8 ± 1.7	3.8 ± 3
2013	2.2 ± 1.8	0.1 ± 0.4	2.8 ± 0.8	0.3 ± 0.8	3.1 ± 1.7	2.9 ± 2.5
2014	2.2 ± 1.9	0.3 ± 1.1	2.9 ± 0.9	0.4 ± 1.8	3.3 ± 2.1	3.6 ± 2.4
2015	2.5 ± 2.0	0.3 ± 0.7	2.9 ± 1.0	0.6 ± 1.3	4.1 ± 1.9	3.4 ± 2.8
2016	2.4 ± 1.8	0.5 ± 0.9	2.8 ± 0.9	0 ± 0	3.7 ± 1.8	3 ± 2.7
2017	2.2 ± 1.6	0.1 ± 0.4	2.8 ± 1	0.3 ± 0.5	3.2 ± 1.1	2.6 ± 2.6
2018	2.6 ± 2.1	1.3 ± 2.4	3 ± 1.2	1 ± 1.1	4.3 ± 2.2	3.6 ± 2.4
Effect sizes	0.263	0.857	0.272	1.2	0.648	0.384
Mean ± SD	2.3 ± 0.2	0.3 ± 0.4	2.8 ± 0.1	0.3 ± 0.3	3.7 ± 0.5	3.2 ± 0.4
Beta-estimator	0.0133	0.079	0.0309	0.0486	0.0297	0.0261
Coefficient of determination	0.0316	0.403	0.529	0.2034	0.0386	0.035
